# Recent insights from non-mammalian models of brain injuries: an emerging literature

**DOI:** 10.3389/fneur.2024.1378620

**Published:** 2024-03-19

**Authors:** Nicole J. Katchur, Daniel A. Notterman

**Affiliations:** ^1^Department of Molecular Biology, Princeton University, Princeton, NJ, United States; ^2^Rutgers-Robert Wood Johnson Medical School, Piscataway, NJ, United States

**Keywords:** repetitive brain injury, non-mammalian models, neurodegeneration, tauopathy, traumatic brain injury

## Abstract

Traumatic brain injury (TBI) is a major global health concern and is increasingly recognized as a risk factor for neurodegenerative diseases including Alzheimer’s disease (AD) and chronic traumatic encephalopathy (CTE). Repetitive TBIs (rTBIs), commonly observed in contact sports, military service, and intimate partner violence (IPV), pose a significant risk for long-term sequelae. To study the long-term consequences of TBI and rTBI, researchers have typically used mammalian models to recapitulate brain injury and neurodegenerative phenotypes. However, there are several limitations to these models, including: (1) lengthy observation periods, (2) high cost, (3) difficult genetic manipulations, and (4) ethical concerns regarding prolonged and repeated injury of a large number of mammals. Aquatic vertebrate model organisms, including *Petromyzon marinus* (sea lampreys), zebrafish (*Danio rerio*), *and invertebrates, Caenorhabditis elegans* (*C. elegans*), and *Drosophila melanogaster* (*Drosophila*), are emerging as valuable tools for investigating the mechanisms of rTBI and tauopathy. These non-mammalian models offer unique advantages, including genetic tractability, simpler nervous systems, cost-effectiveness, and quick discovery-based approaches and high-throughput screens for therapeutics, which facilitate the study of rTBI-induced neurodegeneration and tau-related pathology. Here, we explore the use of non-vertebrate and aquatic vertebrate models to study TBI and neurodegeneration. *Drosophila*, in particular, provides an opportunity to explore the longitudinal effects of mild rTBI and its impact on endogenous tau, thereby offering valuable insights into the complex interplay between rTBI, tauopathy, and neurodegeneration. These models provide a platform for mechanistic studies and therapeutic interventions, ultimately advancing our understanding of the long-term consequences associated with rTBI and potential avenues for intervention.

## 1 Introduction

Traumatic brain injury (TBI) affects an estimated 69 million people each year (1) and impose an economic burden on the world economy of over $400 billion (2). In the United States, more than 472,000 military service members sustained at least one brain injury between 2000 and 2022, with many reporting head injuries before service (3). Studies have shown that TBI is an environmental risk factor for neurodegenerative diseases including Alzheimer’s disease (AD) and other dementias (4, 5) while those with repeated head trauma are at risk of developing chronic traumatic encephalopathy (CTE) (6). A previous head injury increases the risk for a subsequent head injury; thus, greater than 260 per 10,0000 military service members experience subsequent head injury within 1 year of an initial TBI (7). Athletes participating in high-contact sports are at risk for repeated head trauma, and exposure to repetitive TBIs (rTBIs) is common in professional athletes (8, 9). Some American football linemen experience nearly 2,000 impacts over the course of their career (8, 9). While repeated head trauma is commonly linked to contact sports such as football and boxing, it is also evident in the context of intimate partner violence (IPV). Thirty to 94% of women experiencing IPV report at least a single brain injury, with an estimated 80–90% of women sustaining injuries to the head and neck (10, 11). Those who experience brain injuries from IPV may report chronic cognitive impairments in memory and learning (12). Over time, the accumulation of these traumatic events may lead to the development of CTE, a progressive, neurodegenerative disease induced by repeated blows to or rapid displacement of the head, producing chronic changes in cognition, memory, and mood (6). Emerging literature suggests that neurodegenerative changes may occur in women who have experienced IPV, including a recent case study where CTE-like pathology was reported (13, 14). The link between CTE and repeated trauma in athletes is well-established. In a convenience sample of 202 deceased American football players, 87% were diagnosed post-mortem with CTE; the affected percentage was higher (99%) when the sample was restricted to NFL players (15). In a post-mortem study of rugby and soccer players, eleven experienced repeated head trauma, and CTE pathology was found in eight of eleven (16). Despite attempts to use neuroimaging as a mechanism to identify and diagnose CTE before death, the formal diagnosis of CTE occurs only upon autopsy and no effective therapeutic interventions exist to prevent or mitigate neurodegeneration following rTBI.

Mammalian species, such as rats, mice, and pigs, have been used to model TBI and other neurodegenerative diseases including CTE to elucidate long-term outcomes. Although they have provided key insight into numerous secondary injury mechanisms and therapy development (17–19), there are several limitations to the existing literature: (1) lengthy observation periods of the model organism, (2) high cost for experimentation and associated costs, (3) relatively difficult and lengthy genetic manipulations, and (4) ethical concerns regarding a large number of mammals experiencing pain and debilitating injury. For these reasons and others, over the past several decades, researchers have initiated non-mammalian models such as fruit flies (*Drosophila melanogaster; Drosophila*), nematodes (*Caenorhabditis elegans; C. elegans*), zebrafish (*Danio rerio*) and sea lampreys (*Petromyzon marinus*) to model human neurodegenerative diseases and to map the etiopathogenesis of aberrant tau formation after TBI (20). Lower-order vertebrate and invertebrate models offer important potential benefits to studying TBI-induced neurodegeneration, including shorter lifespans to study endpoints, vast genetic tools to manipulate the expression of genes of interest, high-throughput analysis to identify genetic and biochemical networks, screening techniques to identify potential therapeutics, and reduced cost. Here, we highlight the use of non-vertebrate and aquatic vertebrate organisms to define the basic mechanisms underlying repeated TBI and to model rTBI-induced neurodegeneration.

## 2 Mechanisms of acute and repeated traumatic brain injury

### 2.1 Primary injury

As a result of TBI, two separate injuries occur on impact, a primary injury which causes a secondary injury to unfold in the minutes to hours after the initial impact. Blast injury, penetrating injuries, direct impact, and rapid acceleration and deceleration forces can injure the brain, producing a primary injury (21, 22). Within milliseconds, the primary impact produces TBI causing brain tissue to undergo rapid movement and tissue deformation (23). The primary injury leads to the shearing of white matter tracts, resulting in the formation of focal contusions as well as intra-and extracerebral hematomas (22).

In closed-head trauma, mechanical force transmits energy to neurons and glia, which may cause traumatic disruption of CNS structures, disturbances in circulatory autoregulation, impairment of the blood–brain barrier (BBB), and acute cellular dysfunction (23–25). The brain is particularly vulnerable to mechanical force because of its viscoelastic nature and lack of structural support; therefore it is ineffective in withstanding the mechanical forces from a blow to the head (26). Linear acceleration forces exerted during traumatic events can lead to the formation of superficial brain lesions, whereas rotational forces rotate the brain around a fixed axis (27, 28). These rotational forces impart damage to deeper cortical structures (26, 29). Translational forces, specifically linear acceleration forces, impart damage to superficial gray matter, generating cerebral hemorrhages and cortical contusions (27, 30). In contrast, rotational forces mechanically and physiologically damage the deep cerebral white matter axons, resulting in diffuse axonal injury (27, 28). It is hypothesized that axons are further damaged when rapid acceleration and deceleration forces promote the dissociation of tau from microtubules by altering microtubule dynamics, leading to subsequent tau hyperphosphorylation and aggregation (31, 32). However, others suggest that tau hyperphosphorylation occurs first, altering microtubule dynamics, and affecting its association to microtubules (33, 34). Multiple exposures to blast force also result in an accumulation of pathological tau aggregates in the brain (35). Rapid distortion of neuron shape may also induce tau hyperphosphorylation, resulting in tau mislocalization (36). Collectively, these studies suggest that force from a primary injury, at least in part, contributes to the development of neurodegenerative tauopathies.

### 2.2 Secondary injury

The biochemical and cellular responses to the initial impact produce additional damage to the brain, resulting in a secondary injury. Following a primary injury, massive disturbances in brain metabolism, neuroinflammatory responses, microstructural changes, and behavioral changes occur reviewed in (37). Often a consequence of injury, disruption of neuronal and glia osmotic control drives cellular edema, the predominant form of brain edema immediately following TBI (38). Brain edema likely exacerbates injury by increasing cytotoxicity and promoting cell death (39). It is hypothesized that following trauma, extracellular glutamate rises, initiating activation of N-methyl-D-aspartate (NMDA) receptors which promotes the influx of intracellular calcium ions (40). The large influx of calcium activates proteases, endonucleases, and other degradative enzymes and initiates cell death and apoptosis (41).

Oxidative stress damages brain tissue by supplying an excess of reactive oxygen species (ROS) and reactive nitrogen species (RNS) (42). These free radicals disrupt cellular function and preferentially lyse the hydrophobic portion of the lipid bilayer (42). Oxidative stress can oxidize amino acids, resulting in protein modification and loss of catalytic function (43). Protein modifications lead to severe protein aggregation within hours of post-ischemic injury (44). Endogenous antioxidants such as glutathione (GSH) play a vital role in protection against ROS and RNS. Depletion of GSH exacerbates brain infarction following cerebral ischemia (45, 46). After TBI in rodents, GSH decreases in the hippocampus, potentially leading to apoptotic neuronal death (46).

Neuroinflammation, while it can promote recovery during a limited period, also contributes to the pathophysiology of secondary injury by exacerbating damage. The normal BBB prevents the entry of hydrophilic molecules through tight and adherens junctions between endothelial cells (47, 48). Following TBI, the BBB can be disrupted, recruiting leukocytes (49). The damage also activates resident microglial cells, which can remain in an activated state for years following TBI (50, 51). Chronic inflammation following traumatic brain injury increases axonal degeneration and neuronal loss (52, 53), and the resulting injury and brain dysfunction may have a delayed onset and persist long-term, leading to dementia or CTE. Microglia, along with astrocytes, participate in “reactive gliosis,” an aggressive response to neurotrauma involving enlarged glial cells in damaged brain areas (54). Microglial cells function like peripheral macrophages and secrete proinflammatory cytokines and chemokines (55). In both post-brain injury and neurodegenerative disease such as AD, resident immune cells like astrocytes and microglia are elevated (53), implicating inflammation as a potential link between the two phenomena.

Recent evidence suggests that activated microglia can have detrimental effects as they directly correlate with the extent of tau pathology (55, 56) and can increase amyloidogenic amyloid precursor protein (APP) production (57). Cherry and colleagues investigated the relationship between neuroinflammation and CTE and found that the duration of repeated head injury exposure predicted the activated microglial cell density and subsequent greater hyperphosphorylated tau pathology (58). The increase in aberrant APP proliferation eventually leads to the amyloid beta (Aβ) plaques that have been previously associated with AD (59), emphasizing the role of neuroinflammation in the development of continuing injury long after TBI occurs. However, several models of TBI in rodents demonstrate a reduction in amyloid beta plaques following TBI (60, 61), and one study showed that mice overexpressing amyloid precursor protein had a rise in unaggregated Aβ in the hippocampus with extensive hippocampal neuronal death, thereby suggesting that the plaques may be protective against unaggregated (Aβ) toxicity unclear (62), though it remains. Therefore, a more thorough understanding of the complex mechanistic underpinnings of amyloidogenesis and tauopathies must be explored.

### 2.3 Acute and repeated brain trauma

Several studies highlight the different responses to single as opposed to multiple or repeated head injuries by characterizing the immediate and delayed effects on brain metabolism, neuroinflammatory responses, microstructural changes, and behavioral changes. Following a single mild TBI in mice, glucose utilization in the hippocampus and sensorimotor cortex increased in the first 3 days following injury, while rTBI (a second injury 3 days following the first injury) failed to elicit the same immediate response (63). However, after 20 days, rTBI mirrored single head injury with respect to glucose utilization (63), indicating a delayed effect on brain metabolism after rTBI. Moreover, axonal degeneration, increased glial activation and proinflammatory cytokine gene expression were detected 40 days after initial *repeated* injuries, highlighting the prolonged neuroinflammatory responses present after repeated but not single injuries (63). Studies in mammals demonstrate that a single TBI is associated with *transient* increases in hyperphosphorylated tau (64), while depositions of hyperphosphorylated tau aggregates were associated with rTBI (58, 65). Additionally, chronic mild rTBI increased tau abundance within the gray matter up to 3 months following injury (66), and rTBI led to increased phosphorylated tau than a single mild TBI (67). This evidence suggests that acute and repeated injuries have distinct temporal patterns of glucose utilization, neuroinflammatory responses, and tau hyperphosphorylation. Since prolonged neuroinflammatory responses are associated with an increased risk of neurodegenerative disease (68), this evidence suggests particular mechanisms that might be invoked to explain neurodegeneration following repeated, non-disabling head trauma.

Microstructural and behavioral changes also occur after rTBI. Multiple head injuries resulted in more severe microstructural changes, cortical volume loss, behavioral deficits, and histopathological alterations compared to single injuries (69). Jamnia et al. (70) demonstrated persistent memory deficits and structural changes in the cortex and corpus callosum in rats exposed to repeated concussions--three injuries, 48 h apart (70). These rats also experienced deficits in behavior, exhibited anxiety and increased corticosterone levels following rTBI (70). When piglets experienced one high-level rotational injury versus one high-level rotational injury with four subsequent mid-level rotational injuries administered 8 min apart, the multiple rotation injury group experienced greater gait times 1 day post after injury (71). Overall, gait patterns were normal in the single rotation group but were abnormal following the additional rotations (71), suggesting the long-term effect of repeated rotational brain injury on locomotor behavior. Recent studies underscored the accumulating nature of symptoms in adolescents with repeated concussions, with higher symptom scores observed after the second concussion compared to the initial one (72). Following a second concussion, patients reported an increased burden of neuropsychiatric symptoms, particularly in cognitive, sleep, and neuropsychiatric domains (73). *Collectively, these studies emphasize the importance of considering the cumulative effects of repeated head injuries*, with potential long-term consequences on brain structure, function, and behavior.

## 3 Tau’s role in neurodegenerative disease

**Figure 1:**
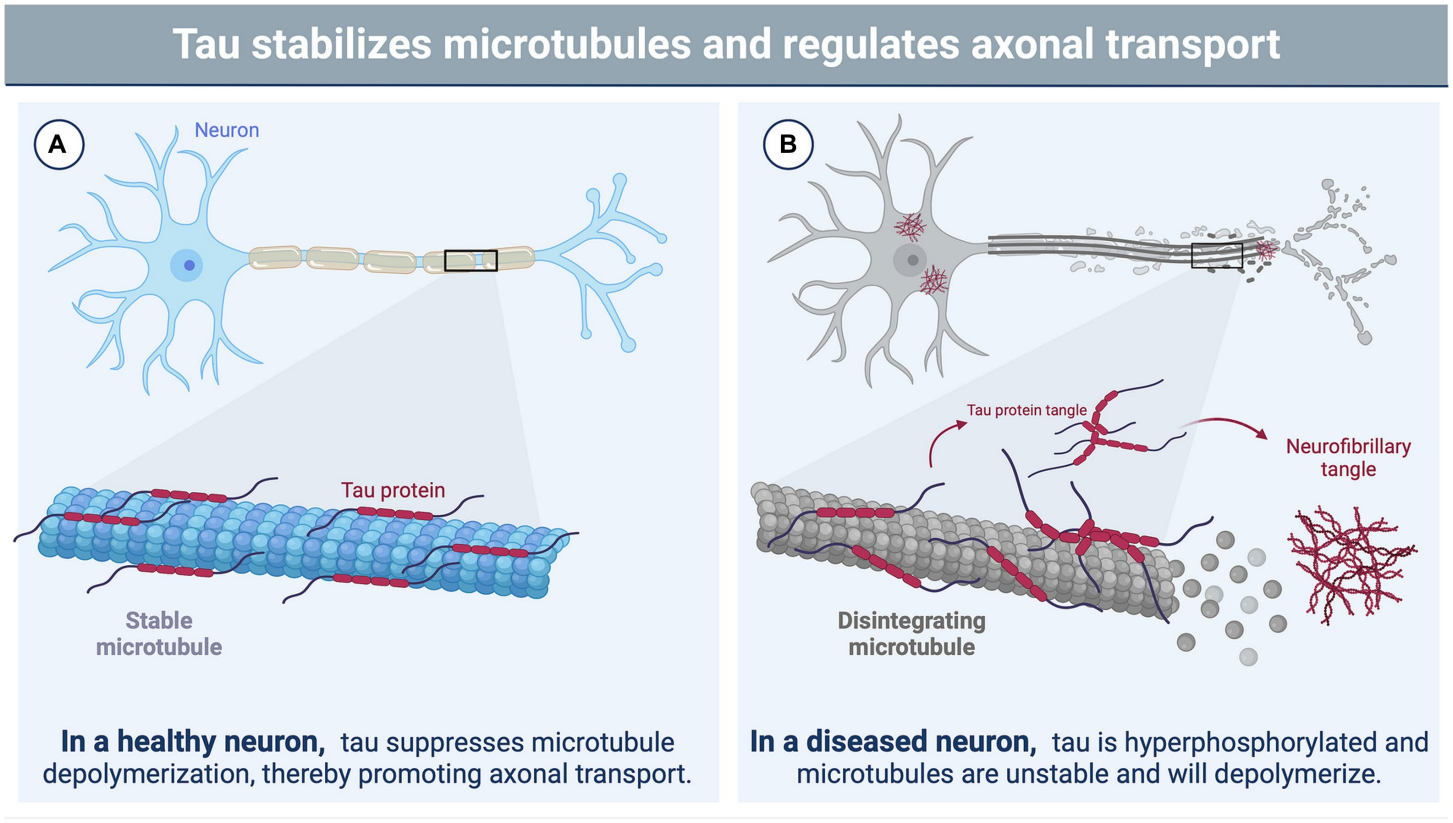
The function of tau in neurons and its role in brain injury. **(A)** In a healthy neuron, tau plays a vital role in stabilizing and supporting axonal transport by binding to microtubules and suppressing microtubule depolymerization (81, 82). The phosphorylation state of tau influences its binding affinity to the microtubule with hypophosphorylation supporting a tighter bind (33, 83, 84). **(B)** Brain injury triggers various cascades, leading to the hyperphosphorylation of tau by protein kinases (85). This hyperphosphorylated state disrupts the binding of tau to microtubules, causing microtubule instability and depolymerization (33, 83, 84). Consequently, tau undergoes filamentous aggregation, forming pathognomonic lesions characteristic of chronic traumatic encephalopathy (CTE), such as neurofibrillary tangles (86). Image created using BioRender.com.

**Figure 2:**
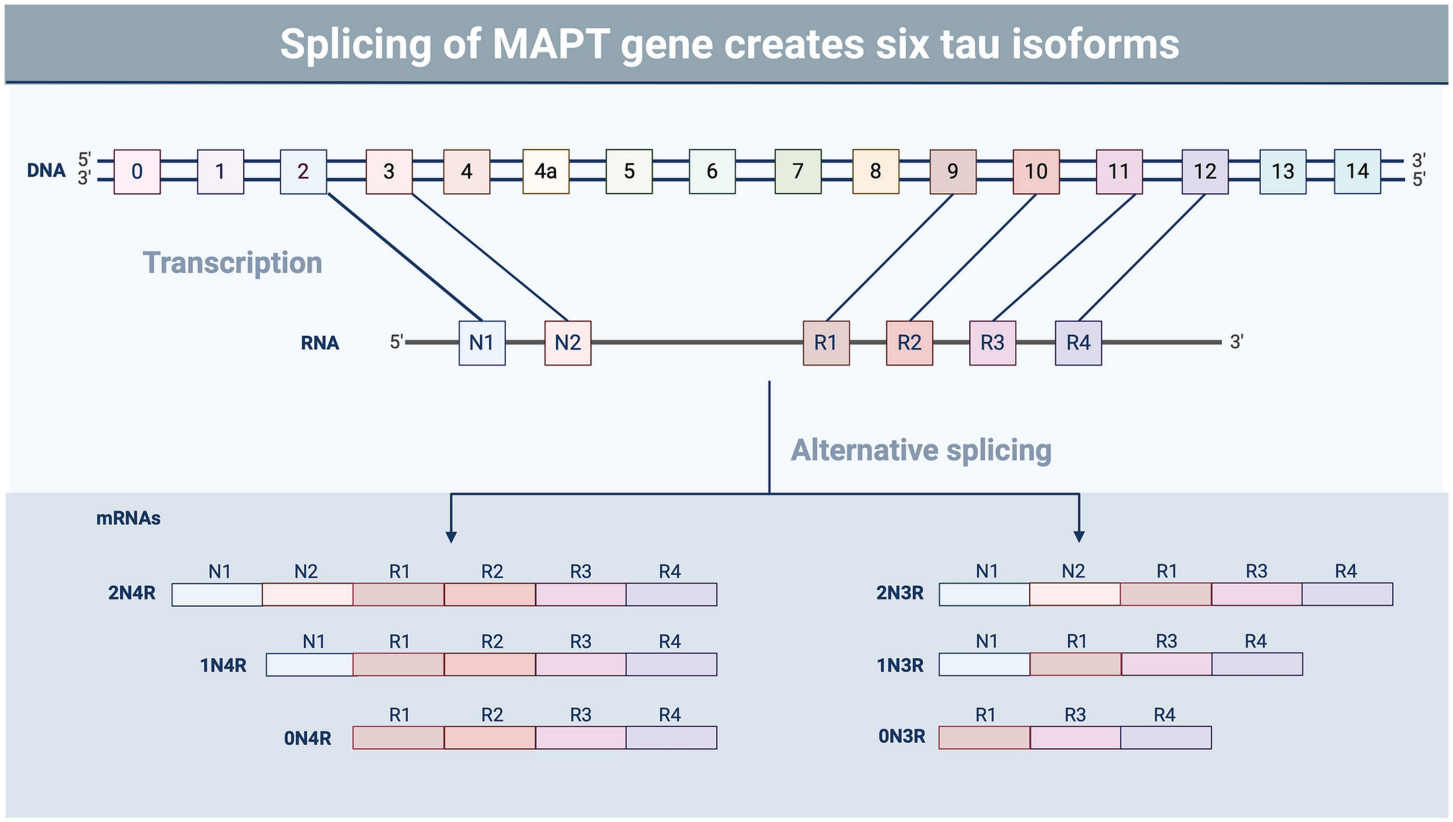
Splicing variants of tau. Tau undergoes alternative splicing involving exons 2, 3, and 10, resulting in six different isoforms of the protein containing the presence or absence of exons containing microtubule-binding domains (R) and N-terminal insertions (N) (87). The ratio of the different tau isoforms varies in different regions of the brain and during different stages of development (88). Tau isoform composition also varies in tauopathic diseases, such as CTE and Alzheimer’s disease, which may impact aggregation and pathology (89). Image created using BioRender.

A recent NINDS consensus document indicated that CTE is likely to occur in the years to decades following rTBI; pathognomonic lesions of tau hyperphosphorylation occur in the cortical sulci surrounding small blood vessels (74). While many areas of the brain may be affected by rTBI, the hippocampus, an important structure for memory and cognition, may be particularly vulnerable to subsequent injuries following a concussion-like injury, leading to changes in mood, memory, and anxiety regulation (75–78). The exact mechanisms by which these cognitive changes are triggered by repeated concussion (rather than physical disruption of neural tissue) remain unclear, though several studies have suggested that neurotoxicity, functional impairment of neuronal synapses, and axonal stabilization by aberrant microtubule-associated protein (MAP) tau may contribute to memory impairment and loss (79, 80) (Figures 1A,B). Tau is a crucial protein in the central nervous system (CNS) involved in the stabilization of microtubules and regulation of axonal transport (81, 82), and its accumulation, hyperphosphorylation, and aberrant localization are recognized as hallmarks of CTE (74). In humans, six different isoforms of tau are produced in the adult brain. These arise via alternative splicing at its amino-and carboxy-terminal ends (Figure 2). Once phosphorylated on multiple sites (e.g., Ser^356^, Ser^396^, Thr^231^), tau loses the ability to bind microtubules (33, 83, 84), thereby promoting microtubule depolymerization and instability.

Abnormal phosphorylation of human tau (hTau) by both non-proline and proline kinases results in insoluble and misfolded tau, leading to the aberrant accumulation and aggregation of filamentous tau polymers, known as paired helical filaments (PHFs) and neurofibrillary tangles (NFTs), two features of CTE (74). Following and perhaps due to the formation of NFTs, neuronal degeneration and death result in release of tau into the extracellular space (90). In turn, this promotes tau uptake into astroglia (91). Some studies have even suggested that the spread of tau through glia cells mirrors a prion-like spread, though whether the misfolded tau actually promotes subsequent local misfolding of the normal *trans* isomer of tau has not been investigated (84, 92).

As noted, the configuration of tau in the *trans* form is the physiological conformation. In contrast, the *cis* conformation of aberrantly phosphorylated tau (p-tau) has been linked to pathogenesis in neurodegenerative disease and of cognitive symptoms (65, 93, 94). In a rodent study of impact and blast injury, the appearance of the *cis* configuration of hyperphosphorylated tau was associated with neurotoxic effects and spread to regions contralateral to the injury, associated with cognitive impairment (65, 94). When targeted with a monoclonal antibody against *cis* p-tau, neuronal apoptosis was prevented, suggesting that accumulation of *cis* p-tau is very early in the pathogenic sequence of post-TBI neurodegeneration (94). PIN1, a peptidyl-prolyl isomerase, plays a role in isomerizing threonine proline bonds at multiple sites (95–97) including those in tau. However, only the isomerization at the phosphorylated Thr^231^-Pro^232^ bond in tau is associated with a biological phenotype (98). The isomerization of p-tau at Thr^231^-Pro^232^ from *cis* to *trans*, promotes both dephosphorylation of tau by PP2A and microtubule stabilization (99). Depletion of Pin1 results in apoptosis and mitotic arrest (100). In an AD model, paired helical filaments contribute to neuronal death (101). Several studies demonstrate that upon restoring the prolyl isomerase in a cell model, Pin1 promotes microtubule binding and stability *in vitro* as well as dephosphorylation at amino acid site Thr^231^ (101, 102). The specific anatomic sites of where tau hyperphosphorylation is found and the pattern of neuronal spread can differentiate between different tauopathic neurodegenerative diseases. In AD, NFTs arise in the brainstem and entorhinal cortex before spreading to the medial temporal lobe and evenly distributing in the neocortex layers III and V (65, 103, 104). In contrast, CTE develops in the deep sulci of the superficial neocortical layers II and III of the cerebral cortex, focally and perivascularly (86). The spread continues irregularly to the neocortex, medial temporal lobe, diencephalon, basal ganglia, and brainstem (65, 105). Though the pathologic spread of tau differs, AD and CTE share at least two of the same tau phosphorylation sites including Thr^231^ and Ser^199^ which have been implicated in neurotoxicity and neuronal dysfunction (65). These common phosphorylation sites, in addition to patterns of deposition, allow AD models of tauopathy to inform CTE tauopathy studies. Throughout this review, “CTE-like” will be used to describe models that recapitulate the phosphorylation and aggregation profile of tau in human CTE, but for which meeting the criterion that tau aggregates occur in the sulci is not possible because the brain is lissencephalic.

Since there is currently no treatment to prevent or mitigate CTE or other forms of neurodegeneration after TBI, researchers have focused on two main drivers of injury-induced sequelae, Pin1 and tau. Lu et al. (101) have produced anti-Pin1 antibodies to restore the function of phosphorylated tau (101) *in vitro* but have not yet extended the studies to *in vivo* CTE-like models. In recent years, the literature has turned its focus to anti-*cis* tau antibodies that work to clear phosphorylated tau plaques in AD, CTE, and severe TBI animal models (93, 94, 106) and have reported improved outcomes *in vivo*. Albayram and colleagues found that repetitive mild injuries led to more severe phosphorylated *cis* tau and tangle-like structures which resemble CTE-like pathology. Treatment with anti-*cis* phosphorylated tau led to the elimination of *cis* phosphorylated tau and total tau accumulation (93). A clinical trial for the use of antibodies targeting *cis*-hyperphosphorylated tau at Thr^231^ is currently underway (107). Perhaps these promising developments in antibody-based therapies will result in effective treatments for CTE and other related tauopathies.

## 4 Non-mammalian models of neurodegenerative disease

For decades, researchers have used rodent models to recapitulate traumatic brain injury and its subsequent sequelae. Difficulties in modeling acceleration and deceleration forces limited the rodent models to specific features of TBI and led to the creation of contusion injury models, namely controlled cortical impact and fluid percussion models (108). The rapid increase in molecular and genetic techniques, in addition to the commercial availability of transgenic rodents and materials, make rodents an attractive substitute for large animal models of brain injury (109). However, large animal models of traumatic brain injury can model a more dynamic range of TBI, namely replicating features of acceleration/deceleration forces (110), which are limited in rodent models. Additionally, rodents have lissencephalic brains with no rigid tentorium cerebelli (109), which restrict the modeling of neurodegenerative diseases with pathology localized to the sulci in the brain. In particular, the pathognomonic lesions of human CTE are found in the sulci near perivascular regions, regions that are particularly vulnerable to mechanical stress from injury (86). Large animal models like primates, have gyrencephalic brains, increasing the translatability of this model to humans (109, 110). Like rodent models, primate models have drawbacks. However, primate models are limited by cost, lack of established post-TBI functional assays, are technically difficult, and raise ethical concerns (109, 110). Therefore, the use of non-mammalian models may be an attractive substitute for large animal and rodent models.

### 4.1 *Petromyzon marinus* (sea lampreys)

Harnessing lower vertebrates for the study of proteins implicated in human disease enables mechanistic studies in a large, identifiable neuron population while extending studies from invertebrates. The robust neuroregenerative capabilities and functional recuperation exhibited by the CNS in lower-order vertebrates make them an attractive experimental model for investigating the role and behavior of abnormal tau following TBI and neurodegeneration. In particular, the biochemical properties of tau have been studied in the lamprey. Hall et al. (111) utilized sea lamprey anterior bulbar cells (ABC) to demonstrate that chronic, full-length human tau overexpression resulted in fibrillary tangles reminiscent of the tau tangles present in neurodegenerative disease, particularly AD (111). They also demonstrated that proprietary small molecules prevented neurodegeneration in cells containing an accumulation of tau filaments (112) while Honson et al. (113) provided evidence for a small molecule inhibitor, N3 (a benzothiazole derivative) to arrest tau aggregate formation in sea lamprey neurons (113). Sea lampreys have also been used to study the movement and deposition of tau and its subsequent role in neurodegeneration. One study demonstrated that mutated tau, particularly the P301L form, migrates in a transneuronal manner, while wild-type tau does not (114, 115). Another study showed that extracellular human tau moves both synaptically and non-synaptically (116). Additionally, exonic mutations in human tau accelerated degeneration in lamprey ABCs (117). Interestingly, Le et al. (116) noted similarities between tau patterns in lampreys and tau patterns in humans. Over time, extracellular tau deposits in the lamprey mirrored the deposits indicative of human CTE and even resembled the perivascular halos that are pathognomonic of CTE in humans. (116). When taken together, these studies suggest that lampreys serve as an excellent model of some features of neurodegenerative disease, highlight its use as a rapid screening tool, and may be used to further investigate the mechanisms driving the formation of aberrant tau after TBI.

### 4.2 *Danio rerio* (zebrafish)

The zebrafish (*Danio rerio*) proteome exhibits a notable degree of homology with the human and they have similar anatomical structures and functions of the brain, thereby making it a suitable organism for investigating TBIs. There are several models that recapitulate closed-headed TBI in the zebrafish. McCutcheon et al. (118) employed a targeted, pulsed, high-intensity focused ultrasound (pHIFU) to induce damage to the brain by mechanical force (118). Zebrafish injured by pHIFU demonstrated increased expression of β-APP and β-III tubulin, a microtubule protein (118), suggesting that this model may be used to investigate the pathophysiology of TBI. Additionally, a non-invasive mild TBI model was developed in adult zebrafish using a laser to induce damage to neural tissue (119). Laser-induced damage to the brain resulted in dilated vessels, hemorrhage and edema, 1 day post-injury (119). These signs suggest that the laser-induced brain injury reproduces features of the pathophysiology associated with mild TBI (119). In the most recent study of zebrafish TBI models, Gill et al. (120) developed a method to model blast injury without the use of anesthetics by dropping a weight onto a fluid-filled plunger (120, 121). This method of injury produced cell death, hemorrhage, blood flow abnormalities, and tauopathy, consistent with TBI (121). The homology between zebrafish and humans in terms of TBI pathophysiology make zebrafish an excellent tool to advance our understanding of TBI and its underlying mechanisms.

In addition to modeling TBI pathophysiology, zebrafish have been used as a biosensor to investigate tauopathies. One study conducted a high-throughput screen for herbal extracts to reduce neuronal death initiated by aberrant tau. Of the 400 herbal extracts screened in the zebrafish, 45 were identified as having the potential to reduce tau-induced neuronal death (122). Additionally, Lopez et al. (123) investigated the clearance kinetics of an aberrant tau protein variant, p.A152T, and applied both pharmacological and genetic approaches to reduce the burden of p.A152T tau in zebrafish by upregulating autophagy (123). Reduction of p.A152T by upregulation of autophagy ameliorated morphological abnormalities and reduced hyperphosphorylated tau (123). In another study, Cosacak et al. (124) created a transgenic zebrafish to explore the aberrant human tau variant, P301L. P301L generates neurofibrillary tangles in mammalian models of tauopathies (125, 126), though did not produce neurofibrillary tangles in the zebrafish nor exacerbate Aβ42 toxicity (124), suggesting a protective mechanism in the zebrafish that may be exploited. These therapies aimed at reducing aberrant tau burden may serve as a strategy for treating tauopathies.

While aquatic vertebrates are useful models to study neurodegeneration and the mechanisms driving aberrant formation of tau, their inherent ability to regenerate neurons (127) following injury may confound the consequences of the secondary injury. However, understanding regeneration may provide key insights into pathways provoked by TBI and may lead to the development of therapeutics to mitigate the effects or potentially reverse TBI-induced pathology. Furthermore, these models provide a way to screen various interventions at relatively low cost while examining histological and biochemical correlates of TBI.

## 5 Invertebrate model organisms

### 5.1 *Caenorhabditis elegans* (roundworms)

In *C. elegans*, researchers have utilized blast injuries to model mild TBI. However, the existing blast methods have yielded heterogeneous outcomes. Angstman et al. developed a shock wave injury model that produces a consistent and quantifiable injury, but its predictive ability for individual outcomes remains limited. However, in 2019, Miansari et al. (128) demonstrated that high-frequency surface acoustic waves (SAW) in a *C. elegans* model of blast-induced mild TBI, confined within a narrow range of the substrate surface, induced mobility and short-term memory deficits in a more homogenous manner than previous models in the literature, suggesting that SAW may be an improved early-stage model for human TBI. Additionally, Angstman et al. have shown that their blast-related model of mild TBI in *C. elegans* recapitulates essential characteristics of human TBI, including loss of consciousness and subsequent recovery (129, 130). This compelling evidence further strengthens the suitability of *C. elegans* as a viable non-mammalian model for TBI and a suitable alternative model organism for mitigating ethical concerns when using mammalian models to explore repetitive trauma.

Beyond inducing injuries, researchers have employed *C. elegans* as a model organism to investigate the effects of TBI-modified tau. Brain homogenates from mice with chronic TBI and or intracerebral inoculation of tau^TBI^, a form of tau that aggregates after chronic TBI, impaired motility, and neuromuscular synaptic transmission in *C. elegans* (131, 132). Surprisingly, when naive mice were intracerebrally inoculated with tau^TBI^, a prion-like spread of tau^TBI^ occurred, resulting in memory deficits and synaptic toxicity (131, 132). Moreover, Diomede et al. established the therapeutic potential of Aβ1-6A2V(D), an all-D-isomer synthetic peptide, to promote tau degradation by proteases and impede tau aggregation in a *C. elegans* model (114). Additionally, the average lifespan of *C. elegans* ranges from 9 to 23 days depending on the rearing temperature (133), highlighting the ability to track tau aggregation through the entire lifespan of the organism. Collectively, these studies demonstrate the potential of using *C. elegans* as biosensors to investigate and manipulate the biochemical properties of tau and its interactions with potential therapeutic peptides in a faster and less complex manner than mammalian models. Overall, *C. elegans* is a valuable alternative to mammalian models for studying neurodegenerative diseases, providing an array of genetic tools and simple mechanistic studies to understand tau in the context of dysfunction, such as TBI.

### 5.2 *Drosophila melanogaster* (fruit flies)

*Drosophila melanogaster* is an excellent model system to study the longitudinal effects of rTBI and its effect on endogenous tau protein. Using *Drosophila,* it is possible to study post-injury behavior, while interrogating histological features of injury and correlating these responses to proteomic and transcriptomic changes (e.g., mass spectroscopy and RNA seq) responses. While tau has been linked to neurodegeneration and neurotoxicity, there is only a rudimentary understanding of the upstream biochemical mediators of tau in the context of rTBI and CTE. Several studies have expressed wild-type and mutant human tau proteins in *Drosophila melanogaster* to model AD, although, *hTau* transgene expression in *Drosophila* is not an ideal functional model, in part because of poor binding to *Drosophila* microtubules. This, as well as differences in phosphorylation sites and uncertainty about whether hTau protein models endogenous NFT formation, limits the applicability of this model. *Drosophila* tau (dTau) contains five putative microtubule-binding repeats and lacks the N-terminal repeats seen in human tau, despite sharing 66% homology with hTau protein (134, 135). At least six CTE-associated phosphorylation sites are observed in human tau, and four of those, Thr^231^, Ser^202^, Thr^205^, Ser^199^ are conserved in dTau, as Thr^151^, Ser^106^, Thr^123^, Ser^103^, respectively. While many studies express hTau in *Drosophila* to model tauopathic diseases, some have shown that dTau can confer the same neurotoxic and neurodegenerative effects as hTau (135). Thus, by investigating dTau in *Drosophila*, its endogenous properties can be readily understood and may represent an informative window into TBI-induced tauopathy (CTE-like) pathogenesis. Overexpression of dTau in *Drosophila* leads to neurotoxicity and eventual neurodegeneration similar to that observed with overexpression of hTau in *Drosophila* (135), though these overexpression models have not been studied in terms of the upstream and downstream mediators of tau-associated neurotoxicity. Neither dTau nor exogenous expression of hTau has been examined with respect to their roles in CTE in *Drosophila*, perhaps due to the lack of sulci and perivascular regions in *Drosophila* that are associated with human CTE pathogenesis. Instead, characterizing and exploring the vulnerable regions in the *Drosophila* brain in the context of repetitive TBI may help to establish a model of chronic traumatic encephalopathy, and evaluating endogenous dTau will provide valuable insights on the progression of tauopathy dysfunction after injury.

The current *Drosophila* models of head-specific TBI study acute changes (136, 137), while current models of rTBI are not head-specific. Traditional methods of TBI in flies utilize high-impact devices (138) or Omni-Bead Ruptor homogenizing platforms (139) that may be used for high-throughput injuries. The high-impact devices utilize a spring attached to a fly vial that, when stretched and released, generates an impact against a tabletop while the Omni-Bead Ruptor freely shakes a small screw cap tube in which the flies are placed. While these methods generate high-throughput injuries, the uncontrolled, full-body injury potentially results in confounding effects on climbing and walking assays, two paradigms commonly used to assess behavioral sequelae after TBI (136). To overcome this limitation, Sun and Chen developed a head-specific model that uses carbon dioxide to propel an impactor against the head (137). They explored walking distance and lifespan as potential markers of injury resulting from repeated head impacts in *Drosophila* (137). Despite this advancement, the use of a manual FlyBuddy switch system introduces variability in the timing of the impacts and the duration in which carbon dioxide propels the impactor. In addition, the underlying neurobiological changes that occur after multiple injuries have not been explored. The Bonini lab developed a fly impactor model using a piezoelectric striker to compress the fly head against a metal fly collar that fixes the head in place and demonstrated acute injury markers that progressed with increasing severity, establishing a more realistic single TBI model in *Drosophila* (136, 140).

## 6 Discussion and conclusion

In this review, we discuss the use of non-vertebrate animal models and vertebrate aquatic animals to explore the mechanisms driving tauopathies and other changes post-TBI. We highlight that these model organisms offer several advantages to research and will allow for cost-effective, rapid, discovery-based approaches and potential high-throughput screens for therapeutics, in addition to reviewing differences in behavioral and physiological response to acute and repeated brain injuries. It is important to note that while acute and repeated injuries differ in effects on glucose metabolism and even in temporal patterns of tau expression, several studies have shown that acute injuries may result in non-transient tau expression (141, 142). In rodents, exposure to a blast injury at 10.8 psi once per day for 3 days resulted in an accumulation of pathological tau aggregates in the brain (35). CTE pathology is also observed in some American football players with multiple concussive and subconcussive blows to the head (143). However, CTE pathology was also found in military personnel who underwent a *single* IED blast injury (143), and a single moderate to severe brain injury resulted in tauopathic lesions in the brain (85). These studies suggest that the total mechanical force accumulated by the brain over time may represent one factor influencing the development of CTE, independent of the number of brain injuries. Severity of the injury, an indirect measure of the mechanical force sustained from a TBI, may also play a role in the development of CTE, with evidence of neuroinflammation in the brain 17 years after the initial injury (50). Several studies demonstrate that chronic neuroinflammation following an acute injury may serve as a contributing factor to neurodegeneration. Given the important role of neuroinflammation in TBI previously discussed and the recent studies that have revealed the powerful effects of microglial depletion strategies on modulating neuroinflammation after TBI, it will be critical to fully characterize the acute and chronic neuroinflammatory responses in a model organism that allows for rapid longitudinal and genetic studies (144–148). The emerging key role of age-related microglial phenotypes, recently described by (145), in this regard, and their link to neurodegeneration could represent a perfect opportunity for exploration in TBI models in *Drosophila*, given the relative ease and efficiency to study long-term effects and outcomes.

## Author contributions

NK: Conceptualization, Writing – original draft, Writing – review & editing. DN: Supervision, Writing – original draft, Writing – review & editing.

## References

[1] Dewan MC Rattani A Gupta S Baticulon RE Hung Y-C Punchak M. Estimating the global incidence of traumatic brain injury. J Neurosurg. (2018) 130:1080–97. doi: 10.3171/2017.10.JNS17352 29701556

[2] Maas AIR Menon DK Adelson PD Andelic N Bell MJ Belli A. Traumatic brain injury: integrated approaches to improve prevention, clinical care, and research. Lancet Neurol. (2017) 16:987–1048. doi: 10.1016/S1474-4422(17)30371-X 29122524

[3] DOD TBI Worldwide Numbers (2023). Military health system. Available at: https://health.mil/Military-Health-Topics/Centers-of-Excellence/Traumatic-Brain-Injury-Center-of-Excellence/DOD-TBI-Worldwide-Numbers (Accessed July 24, 2023).

[4] Kenney K Iacono D Edlow BL Katz DI Diaz-Arrastia R Dams-O’Connor K. Dementia after moderate-severe traumatic brain injury: coexistence of multiple Proteinopathies. J Neuropathol Exp Neurol. (2018) 77:50–63. doi: 10.1093/jnen/nlx101, PMID: 29155947 PMC5939622

[5] Mortimer JA van Duijn CM Chandra V Fratiglioni L Graves AB Heyman A. Head trauma as a risk factor for Alzheimer’s disease: a collaborative re-analysis of case-control studies. EURODEM risk factors research group. Int J Epidemiol. (1991) 20 Suppl 2:S28–35. doi: 10.1093/ije/20.Supplement_2.S28, PMID: 1833351

[6] McKee AC Stein TD Huber BR Crary JF Bieniek K Dickson D. Chronic traumatic encephalopathy (CTE): criteria for neuropathological diagnosis and relationship to repetitive head impacts. Acta Neuropathol. (2023) 145:371–94. doi: 10.1007/s00401-023-02540-w, PMID: 36759368 PMC10020327

[7] Agimi Y Earyes L Deressa T Stout K. Estimating repeat traumatic brain injury in the U.S. military, 2015-2017. Mil Med. (2022) 187:e360–7. doi: 10.1093/milmed/usab041, PMID: 33591307

[8] Crisco JJ Fiore R Beckwith JG Chu JJ Brolinson PG Duma S. Frequency and location of head impact exposures in individual collegiate football players. J Athl Train. (2010) 45:549–59. doi: 10.4085/1062-6050-45.6.549, PMID: 21062178 PMC2978006

[9] Stern RA Riley DO Daneshvar DH Nowinski CJ Cantu RC McKee AC. Long-term consequences of repetitive brain trauma: chronic traumatic encephalopathy. PM R. (2011) 3:S460–7. doi: 10.1016/j.pmrj.2011.08.008, PMID: 22035690

[10] Adhikari SP Maldonado-Rodriguez N Smiley SC Lewis CD Horst MD Jeffrey Lai CW. Characterizing possible acute brain injury in women experiencing intimate partner violence: a retrospective chart review. Violence Against Women. (2023) 10778012231159417:107780122311594. doi: 10.1177/10778012231159417 PMC11292972 36855801

[11] Kwako LE Glass N Campbell J Melvin KC Barr T Gill JM. Traumatic brain injury in intimate partner violence: a critical review of outcomes and mechanisms. Trauma Violence Abuse. (2011) 12:115–26. doi: 10.1177/1524838011404251, PMID: 21511686

[12] Costello K Greenwald BD. Update on domestic violence and traumatic brain injury: a narrative review. Brain Sci. (2022) 12. doi: 10.3390/brainsci12010122 PMC8773525 35053865

[13] Ayton D Pritchard E Tsindos T. Acquired brain injury in the context of family violence: a systematic scoping review of incidence, prevalence, and contributing factors. Trauma Violence Abuse. (2021) 22:3–17. doi: 10.1177/1524838018821951, PMID: 30651050

[14] Danielsen T Hauch C Kelly L White CL. Chronic traumatic encephalopathy (CTE)-type neuropathology in a Young victim of domestic abuse. J Neuropathol Exp Neurol. (2021) 80:624–7. doi: 10.1093/jnen/nlab015, PMID: 33706376

[15] Mez J Daneshvar DH Kiernan PT Abdolmohammadi B Alvarez VE Huber BR. Clinicopathological evaluation of chronic traumatic encephalopathy in players of American football. JAMA. (2017) 318:360–70. doi: 10.1001/jama.2017.8334, PMID: 28742910 PMC5807097

[16] Lee EB Kinch K Johnson VE Trojanowski JQ Smith DH Stewart W. Chronic traumatic encephalopathy is a common co-morbidity, but less frequent primary dementia in former soccer and rugby players. Acta Neuropathol. (2019) 138:389–99. doi: 10.1007/s00401-019-02030-y, PMID: 31152201 PMC6689293

[17] DeWitt DS Hawkins BE Dixon CE Kochanek PM Armstead W Bass CR. Pre-clinical testing of therapies for traumatic brain injury. J Neurotrauma. (2018) 35:2737–54. doi: 10.1089/neu.2018.5778, PMID: 29756522 PMC8349722

[18] Kochanek PM Dixon CE Mondello S Wang KKK Lafrenaye A Bramlett HM. Multi-center pre-clinical consortia to enhance translation of therapies and biomarkers for traumatic brain injury: operation brain trauma therapy and beyond. Front Neurol. (2018) 9:640. doi: 10.3389/fneur.2018.00640, PMID: 30131759 PMC6090020

[19] Smith DH Hicks R Povlishock JT. Therapy development for diffuse axonal injury. J Neurotrauma. (2013) 30:307–23. doi: 10.1089/neu.2012.2825, PMID: 23252624 PMC3627407

[20] Zulazmi NA Arulsamy A Ali I Zainal Abidin SA Othman I Shaikh MF. The utilization of small non-mammals in traumatic brain injury research: a systematic review. CNS Neurosci Ther. (2021) 27:381–402. doi: 10.1111/cns.13590, PMID: 33539662 PMC7941175

[21] Dixon KJ. Pathophysiology of traumatic brain injury. Phys Med Rehabil Clin N Am. (2017) 28:215–25. doi: 10.1016/j.pmr.2016.12.001 28390509

[22] Maas AIR Stocchetti N Bullock R. Moderate and severe traumatic brain injury in adults. Lancet Neurol. (2008) 7:728–41. doi: 10.1016/S1474-4422(08)70164-9 18635021

[23] Wang F Han Y Wang B Peng Q Huang X Miller K. Prediction of brain deformations and risk of traumatic brain injury due to closed-head impact: quantitative analysis of the effects of boundary conditions and brain tissue constitutive model. Biomech Model Mechanobiol. (2018) 17:1165–85. doi: 10.1007/s10237-018-1021-z, PMID: 29754317

[24] Logsdon AF Lucke-Wold BP Turner RC Huber JD Rosen CL Simpkins JW. Role of microvascular disruption in brain damage from traumatic brain injury. Compr Physiol. (2015) 5:1147–60. doi: 10.1002/cphy.c140057, PMID: 26140712 PMC4573402

[25] McAllister TW. Neurobiological consequences of traumatic brain injury. Dialogues Clin Neurosci. (2011) 13:287–300. doi: 10.31887/DCNS.2011.13.2/tmcallister, PMID: 22033563 PMC3182015

[26] McAllister TW Stein MB. Effects of psychological and biomechanical trauma on brain and behavior. Ann N Y Acad Sci. (2010) 1208:46–57. doi: 10.1111/j.1749-6632.2010.05720.x, PMID: 20955325 PMC3169086

[27] Greve MW Zink BJ. Pathophysiology of traumatic brain injury. Mt Sinai J Med. (2009) 76:97–104. doi: 10.1002/msj.20104 19306379

[28] Johnson VE Stewart W Smith DH. Axonal pathology in traumatic brain injury. Exp Neurol. (2013) 246:35–43. 22285252 10.1016/j.expneurol.2012.01.013 PMC3979341

[29] Runnerstam M Bao F Huang Y Shi J Gutierrez E Hamberger A. A new model for diffuse brain injury by rotational acceleration: II. Effects on extracellular glutamate, intracranial pressure, and neuronal apoptosis. J Neurotrauma. (2001) 18:259–73. doi: 10.1089/08977150151070892, PMID: 11284547

[30] Ommaya AK Goldsmith W Thibault L. Biomechanics and neuropathology of adult and paediatric head injury. Br J Neurosurg. (2002) 16:220–42. doi: 10.1080/02688690220148824 12201393

[31] Duquette A Pernègre C Veilleux Carpentier A Leclerc N. Similarities and differences in the pattern of tau hyperphosphorylation in physiological and pathological conditions: impacts on the elaboration of therapies to prevent tau pathology. Front Neurol. (2020) 11:607680. doi: 10.3389/fneur.2020.607680 33488502 PMC7817657

[32] Tagge CA Fisher AM Minaeva OV Gaudreau-Balderrama A Moncaster JA Zhang X-L. Concussion, microvascular injury, and early tauopathy in young athletes after impact head injury and an impact concussion mouse model. Brain. (2018) 141:422–58. doi: 10.1093/brain/awx350, PMID: 29360998 PMC5837414

[33] Lasagna-Reeves CA Castillo-Carranza DL Sengupta U Sarmiento J Troncoso J Jackson GR. Identification of oligomers at early stages of tau aggregation in Alzheimer’s disease. FASEB J. (2012) 26:1946–59. PMID: 22253473 10.1096/fj.11-199851 PMC4046102

[34] Liu F Li B Tung E-J Grundke-Iqbal I Iqbal K Gong C-X. Site-specific effects of tau phosphorylation on its microtubule assembly activity and self-aggregation. Eur J Neurosci. (2007) 26:3429–36. doi: 10.1111/j.1460-9568.2007.05955.x, PMID: 18052981 PMC2262108

[35] Dickstein DL De Gasperi R Gama Sosa MA Perez-Garcia G Short JA Sosa H. Brain and blood biomarkers of tauopathy and neuronal injury in humans and rats with neurobehavioral syndromes following blast exposure. Mol Psychiatry. (2021) 26:5940–54. doi: 10.1038/s41380-020-0674-z, PMID: 32094584 PMC7484380

[36] Braun NJ Yao KR Alford PW Liao D. Mechanical injuries of neurons induce tau mislocalization to dendritic spines and tau-dependent synaptic dysfunction. Proc Natl Acad Sci USA. (2020) 117:29069–79. doi: 10.1073/pnas.2008306117, PMID: 33139536 PMC7682580

[37] Hashimoto Y Kinoshita N Greco TM Federspiel JD Jean Beltran PM Ueno N. Mechanical force induces phosphorylation-mediated signaling that underlies tissue response and robustness in Xenopus embryos. Cell Syst. (2019) 8:226–241.e7. doi: 10.1016/j.cels.2019.01.006, PMID: 30852251 PMC6453581

[38] Marmarou A. A review of progress in understanding the pathophysiology and treatment of brain edema. Neurosurg Focus. (2007) 22:E1–E10. doi: 10.3171/foc.2007.22.5.2, PMID: 17613227

[39] Jha RM Kochanek PM Simard JM. Pathophysiology and treatment of cerebral edema in traumatic brain injury. Neuropharmacology. (2019) 145:230–46. doi: 10.1016/j.neuropharm.2018.08.004, PMID: 30086289 PMC6309515

[40] Krishnamurthy K Laskowitz DT. Cellular and molecular mechanisms of secondary neuronal injury following traumatic brain injury In: Laskowitz D Grant G, editors. Translational research in traumatic brain injury. Boca Raton, FL: CRC Press/Taylor and Francis Group (n.d.) 26583177

[41] Zhivotovsky B Orrenius S. Calcium and cell death mechanisms: a perspective from the cell death community. Cell Calcium. (2011) 50:211–21. doi: 10.1016/j.ceca.2011.03.003, PMID: 21459443

[42] Shohami E. Kohen R. “The role of reactive oxygen species in the pathogenesis of traumatic brain injury,” in: Natan G Hans H, editors. Göbel for Oxidative Stress and Free Radical Damage in Neurology. Totowa, NJ: Humana Press, (2011) 99–118.

[43] Sohal RS. Role of oxidative stress and protein oxidation in the aging process. Free Radic Biol Med. (2002) 33:37–44. doi: 10.1016/S0891-5849(02)00856-0, PMID: 12086680

[44] Kahl A Blanco I Jackman K Baskar J Milaganur Mohan H Rodney-Sandy R. Cerebral ischemia induces the aggregation of proteins linked to neurodegenerative diseases. Sci Rep. (2018) 8:2701. doi: 10.1038/s41598-018-21063-z, PMID: 29426953 PMC5807442

[45] Ansari MA Roberts KN Scheff SW. Oxidative stress and modification of synaptic proteins in hippocampus after traumatic brain injury. Free Radic Biol Med. (2008) 45:443–52. doi: 10.1016/j.freeradbiomed.2008.04.038, PMID: 18501200 PMC2586827

[46] Mizui T Kinouchi H Chan PH. Depletion of brain glutathione by buthionine sulfoximine enhances cerebral ischemic injury in rats. Am J Phys. (1992) 262:H313–7. doi: 10.1152/ajpheart.1992.262.2.H313, PMID: 1539690

[47] Daneman R Prat A. The blood-brain barrier. Cold Spring Harb Perspect Biol. (2015) 7:a020412. doi: 10.1101/cshperspect.a020412, PMID: 25561720 PMC4292164

[48] Sulhan S Lyon KA Shapiro LA Huang JH. Neuroinflammation and blood-brain barrier disruption following traumatic brain injury: pathophysiology and potential therapeutic targets. J Neurosci Res. (2020) 98:19–28. doi: 10.1002/jnr.24331, PMID: 30259550 PMC6437022

[49] Winkler EA Minter D Yue JK Manley GT. Cerebral edema in traumatic brain injury: pathophysiology and prospective therapeutic targets. Neurosurg Clin N Am. (2016) 27:473–88. doi: 10.1016/j.nec.2016.05.008 27637397

[50] Ramlackhansingh AF Brooks DJ Greenwood RJ Bose SK Turkheimer FE Kinnunen KM. Inflammation after trauma: microglial activation and traumatic brain injury. Ann Neurol. (2011) 70:374–83. doi: 10.1002/ana.22455 21710619

[51] Smith C Gentleman SM Leclercq PD Murray LS Griffin WST Graham DI. The neuroinflammatory response in humans after traumatic brain injury. Neuropathol Appl Neurobiol. (2013) 39:654–66. doi: 10.1111/nan.12008, PMID: 23231074 PMC3833642

[52] Ertürk A Mentz S Stout EE Hedehus M Dominguez SL Neumaier L. Interfering with the chronic immune response rescues chronic degeneration after traumatic brain injury. J Neurosci. (2016) 36:9962–75. doi: 10.1523/JNEUROSCI.1898-15.2016, PMID: 27656033 PMC6705567

[53] Kokiko-Cochran ON Godbout JP. The inflammatory continuum of traumatic brain injury and Alzheimer’s disease. Front Immunol. (2018) 9:672. doi: 10.3389/fimmu.2018.00672, PMID: 29686672 PMC5900037

[54] Verkhratsky A Butt A Li B Illes P Zorec R Semyanov A. Astrocytes in human central nervous system diseases: a frontier for new therapies. Signal Transduct Target Ther. (2023) 8:396. doi: 10.1038/s41392-023-01628-9, PMID: 37828019 PMC10570367

[55] Ziebell JM Morganti-Kossmann MC. Involvement of pro-and anti-inflammatory cytokines and chemokines in the pathophysiology of traumatic brain injury. Neurotherapeutics. (2010) 7:22–30. doi: 10.1016/j.nurt.2009.10.016, PMID: 20129494 PMC5084109

[56] Odfalk KF Bieniek KF Hopp SC. Microglia: Friend and foe in tauopathy. Prog Neurobiol. (2022) 216:102306. doi: 10.1016/j.pneurobio.2022.102306, PMID: 35714860 PMC9378545

[57] Griffin WS Sheng JG Gentleman SM Graham DI Mrak RE Roberts GW. Microglial interleukin-1 alpha expression in human head injury: correlations with neuronal and neuritic beta-amyloid precursor protein expression. Neurosci Lett. (1994) 176:133–6. doi: 10.1016/0304-3940(94)90066-3, PMID: 7830934 PMC3833643

[58] Cherry JD Tripodis Y Alvarez VE Huber B Kiernan PT Daneshvar DH. Microglial neuroinflammation contributes to tau accumulation in chronic traumatic encephalopathy. Acta Neuropathol Commun. (2016) 4:112. doi: 10.1186/s40478-016-0382-8, PMID: 27793189 PMC5084333

[59] Kirouac L Rajic AJ Cribbs DH Padmanabhan J. Activation of Ras-ERK signaling and GSK-3 by amyloid precursor protein and amyloid Beta facilitates neurodegeneration in Alzheimer’s disease. eNeuro. (2017) 4:ENEURO.0149–16.2017. doi: 10.1523/ENEURO.0149-16.2017, PMID: 28374012 PMC5367084

[60] Johnson VE Stewart W Smith DH. Traumatic brain injury and amyloid-β pathology: a link to Alzheimer’s disease? Nat Rev Neurosci. (2010) 11:361–70. doi: 10.1038/nrn2808, PMID: 20216546 PMC3979339

[61] Szczygielski J Mautes A Steudel WI Falkai P Bayer TA Wirths O. Traumatic brain injury: cause or risk of Alzheimer’s disease? A review of experimental studies. J Neural Transm. (2005) 112:1547–64. doi: 10.1007/s00702-005-0326-0 15959838

[62] Smith DH Nakamura M McIntosh TK Wang J Rodríguez A Chen XH. Brain trauma induces massive hippocampal neuron death linked to a surge in beta-amyloid levels in mice overexpressing mutant amyloid precursor protein. Am J Pathol. (1998) 153:1005–10. doi: 10.1016/S0002-9440(10)65643-X, PMID: 9736050 PMC1853010

[63] Weil ZM Gaier KR Karelina K. Injury timing alters metabolic, inflammatory and functional outcomes following repeated mild traumatic brain injury. Neurobiol Dis. (2014) 70:108–16. doi: 10.1016/j.nbd.2014.06.016, PMID: 24983210

[64] Mouzon B Bachmeier C Ojo J Acker C Ferguson S Crynen G. Chronic White matter degeneration, but no tau pathology at one-year post-repetitive mild traumatic brain injury in a tau transgenic model. J Neurotrauma. (2019) 36:576–88. doi: 10.1089/neu.2018.5720, PMID: 29993324 PMC6354615

[65] Katsumoto A Takeuchi H Tanaka F. Tau pathology in chronic traumatic encephalopathy and Alzheimer’s disease: similarities and differences. Front Neurol. (2019) 10:980. doi: 10.3389/fneur.2019.00980, PMID: 31551922 PMC6748163

[66] Ojo JO Mouzon B Algamal M Leary P Lynch C Abdullah L. Chronic repetitive mild traumatic brain injury results in reduced cerebral blood flow, axonal injury, gliosis, and increased T-tau and tau oligomers. J Neuropathol Exp Neurol. (2016) 75:636–55. doi: 10.1093/jnen/nlw035, PMID: 27251042 PMC4913432

[67] Ojo JO Mouzon B Greenberg MB Bachmeier C Mullan M Crawford F. Repetitive mild traumatic brain injury augments tau pathology and glial activation in aged hTau mice. J Neuropathol Exp Neurol. (2013) 72:137–51. doi: 10.1097/NEN.0b013e3182814cdf, PMID: 23334597

[68] Chen W-W Zhang X Huang W-J. Role of neuroinflammation in neurodegenerative diseases (review). Mol Med Rep. (2016) 13:3391–6. doi: 10.3892/mmr.2016.4948, PMID: 26935478 PMC4805095

[69] Kao Y-CJ Lui YW Lu C-F Chen H-L Hsieh B-Y Chen C-Y. Behavioral and structural effects of single and repeat closed-head injury. AJNR Am J Neuroradiol. (2019) 40:601–8. doi: 10.3174/ajnr.A6014 30923084 PMC6945510

[70] Jamnia N Urban JH Stutzmann GE Chiren SG Reisenbigler E Marr R. A clinically relevant closed-head model of single and repeat concussive injury in the adult rat using a controlled cortical impact device. J Neurotrauma. (2017) 34:1351–63. doi: 10.1089/neu.2016.4517, PMID: 27762651

[71] Mull M Aderibigbe O Hajiaghamemar M Oeur RA Margulies SS. Multiple head rotations result in persistent gait alterations in piglets. Biomedicines. (2022) 10:2976. doi: 10.3390/biomedicines10112976, PMID: 36428544 PMC9687234

[72] Wilson RJ Bell MR Giordano KR Seyburn S Kozlowski DA. Repeat subconcussion in the adult rat gives rise to behavioral deficits similar to a single concussion but different depending upon sex. Behav Brain Res. (2023) 438:114206. doi: 10.1016/j.bbr.2022.114206, PMID: 36356721

[73] Quinones A Young T Schupper AJ Ali M Hrabarchuk EI Lamb CD. Effects of repetitive head trauma on symptomatology of subsequent sport-related concussion. J Neurosurg Pediatr. (2023) 32:133–40. doi: 10.3171/2023.2.PEDS237, PMID: 37161993

[74] McKee AC Cairns NJ Dickson DW Folkerth RD Keene CD Litvan I. The first NINDS/NIBIB consensus meeting to define neuropathological criteria for the diagnosis of chronic traumatic encephalopathy. Acta Neuropathol. (2016) 131:75–86. doi: 10.1007/s00401-015-1515-z, PMID: 26667418 PMC4698281

[75] Effgen G. B. Ong T. Nammalwar S. Ortuño A. I. Meaney D. F., ‘Dale' Bass, C. R., . Primary blast exposure increases hippocampal vulnerability to subsequent exposure: reducing Long-term potentiation. J Neurotrauma. (2016) 33:1901–12. doi: 10.1089/neu.2015.4327, PMID: 26699926 PMC6445278

[76] Meyer DL Davies DR Barr JL Manzerra P Forster GL. Mild traumatic brain injury in the rat alters neuronal number in the limbic system and increases conditioned fear and anxiety-like behaviors. Exp Neurol. (2012) 235:574–87. doi: 10.1016/j.expneurol.2012.03.012, PMID: 22498103

[77] Nawashiro H Shima K Chigasaki H. Selective vulnerability of hippocampal CA3 neurons to hypoxia after mild concussion in the rat. Neurol Res. (1995) 17:455–60. doi: 10.1080/01616412.1995.11740363, PMID: 8622802

[78] Tang Y-P Noda Y Hasegawa T Nabeshima T. A concussive-like brain injury model in mice (II): selective neuronal loss in the cortex and Hippocampus. J Neurotrauma. (1997) 14:863–73. doi: 10.1089/neu.1997.14.863, PMID: 9421457

[79] Cheng JS Craft R Yu G-Q Ho K Wang X Mohan G. Tau reduction diminishes spatial learning and memory deficits after mild repetitive traumatic brain injury in mice. PLoS One. (2014) 9:e115765. doi: 10.1371/journal.pone.0115765, PMID: 25551452 PMC4281043

[80] Schindowski K Bretteville A Leroy K Bégard S Brion J-P Hamdane M. Alzheimer’s disease-like tau neuropathology leads to memory deficits and loss of functional synapses in a novel mutated tau transgenic mouse without any motor deficits. Am J Pathol. (2006) 169:599–616. doi: 10.2353/ajpath.2006.060002, PMID: 16877359 PMC1698785

[81] Feinstein SC Wilson L. Inability of tau to properly regulate neuronal microtubule dynamics: a loss-of-function mechanism by which tau might mediate neuronal cell death. Biochim Biophys Acta (BBA) - Mol Basis Dis. (2005) 1739:268–79. doi: 10.1016/j.bbadis.2004.07.002 15615645

[82] Wang J-Z Liu F. Microtubule-associated protein tau in development, degeneration and protection of neurons. Prog Neurobiol. (2008) 85:148–75. doi: 10.1016/j.pneurobio.2008.03.002 18448228

[83] Alonso AC Mederlyova A Novak M Grundke-Iqbal I Iqbal K. Promotion of hyperphosphorylation by frontotemporal dementia tau mutations. J Biol Chem. (2004) 279:34873–81. doi: 10.1074/jbc.M405131200 15190058

[84] Xia Y Bell BM Kim JD Giasson BI. Tau mutation S356T in the three repeat isoform leads to microtubule dysfunction and promotes prion-like seeded aggregation. Front Neurosci. (2023) 17:1181804. doi: 10.3389/fnins.2023.1181804, PMID: 37304025 PMC10248064

[85] Johnson VE Stewart W Smith DH. Widespread tau and amyloid-Beta pathology many years after a single traumatic brain injury in humans. Brain Pathol. (2012) 22:142–9. doi: 10.1111/j.1750-3639.2011.00513.x, PMID: 21714827 PMC3979351

[86] McKee AC Stein TD Kiernan PT Alvarez VE. The neuropathology of chronic traumatic encephalopathy. Brain Pathol. (2015) 25:350–64. doi: 10.1111/bpa.12248, PMID: 25904048 PMC4526170

[87] Andreadis A. Tau gene alternative splicing: expression patterns, regulation and modulation of function in normal brain and neurodegenerative diseases. Biochim Biophys Acta. (2005) 1739:91–103. doi: 10.1016/j.bbadis.2004.08.010, PMID: 15615629

[88] Majounie E Cross W Newsway V Dillman A Vandrovcova J Morris CM. Variation in tau isoform expression in different brain regions and disease states. Neurobiol Aging. (2013) 34:1922.e7–1922.e12. doi: 10.1016/j.neurobiolaging.2013.01.017, PMID: 23428180 PMC3642280

[89] Kovacs GG Ghetti B Goedert M. Classification of diseases with accumulation of tau protein. Neuropathol Appl Neurobiol. (2022) 48:e12792. doi: 10.1111/nan.12792, PMID: 35064600 PMC9352145

[90] Metaxas A Kempf SJ. Neurofibrillary tangles in Alzheimer’s disease: elucidation of the molecular mechanism by immunohistochemistry and tau protein phospho-proteomics. Neural Regeneration Res. (2016) 11:1579–81. doi: 10.4103/1673-5374.193234 PMC5116834 27904486

[91] Piacentini R Li Puma DD Mainardi M Lazzarino G Tavazzi B Arancio O. Reduced gliotransmitter release from astrocytes mediates tau-induced synaptic dysfunction in cultured hippocampal neurons. Glia. (2017) 65:1302–16. doi: 10.1002/glia.23163, PMID: 28519902 PMC5520670

[92] Vasconcelos B Stancu I-C Buist A Bird M Wang P Vanoosthuyse A. Heterotypic seeding of tau fibrillization by pre-aggregated Abeta provides potent seeds for prion-like seeding and propagation of tau-pathology in vivo. Acta Neuropathol. (2016) 131:549–69. doi: 10.1007/s00401-015-1525-x, PMID: 26739002 PMC4789256

[93] Albayram O Kondo A Mannix R Smith C Tsai C-Y Li C. Cis P-tau is induced in clinical and preclinical brain injury and contributes to post-injury sequelae. Nat Commun. (2017) 8:1000. doi: 10.1038/s41467-017-01068-4, PMID: 29042562 PMC5645414

[94] Kondo A Shahpasand K Mannix R Qiu J Moncaster J Chen C-H. Antibody against early driver of neurodegeneration cis P-tau blocks brain injury and tauopathy. Nature. (2015) 523:431–6. doi: 10.1038/nature14658, PMID: 26176913 PMC4718588

[95] Kimura T Tsutsumi K Taoka M Saito T Masuda-Suzukake M Ishiguro K. Isomerase Pin1 stimulates dephosphorylation of tau protein at cyclin-dependent kinase (Cdk5)-dependent Alzheimer phosphorylation sites. J Biol Chem. (2013) 288:7968–77. doi: 10.1074/jbc.M112.433326 23362255 PMC3597833

[96] Smet C Sambo A-V Wieruszeski J-M Leroy A Landrieu I Buée L. The peptidyl prolyl cis/trans-isomerase Pin1 recognizes the phospho-Thr212-Pro213 site on tau. Biochemistry. (2004) 43:2032–40. doi: 10.1021/bi035479x, PMID: 14967043

[97] Smet C Wieruszeski J-M Buée L Landrieu I Lippens G. Regulation of Pin1 peptidyl-prolyl cis/trans isomerase activity by its WW binding module on a multi-phosphorylated peptide of tau protein. FEBS Lett. (2005) 579:4159–64. doi: 10.1016/j.febslet.2005.06.048, PMID: 16024016

[98] Nakamura K Greenwood A Binder L Bigio EH Denial S Nicholson L. Proline isomer-specific antibodies reveal the early pathogenic tau conformation in Alzheimer’s disease. Cell. (2012) 149:232–44. doi: 10.1016/j.cell.2012.02.016, PMID: 22464332 PMC3601591

[99] Albayram O Herbert MK Kondo A Tsai C-Y Baxley S Lian X. Function and regulation of tau conformations in the development and treatment of traumatic brain injury and neurodegeneration. Cell Biosci. (2016) 6:59. doi: 10.1186/s13578-016-0124-4, PMID: 27980715 PMC5139118

[100] Lu KP Hanes SD Hunter T. A human peptidyl–prolyl isomerase essential for regulation of mitosis. Nature. (1996) 380:544–7. doi: 10.1038/380544a0, PMID: 8606777

[101] Lu PJ Wulf G Zhou XZ Davies P Lu KP. The prolyl isomerase Pin1 restores the function of Alzheimer-associated phosphorylated tau protein. Nature. (1999) 399:784–8. doi: 10.1038/21650, PMID: 10391244

[102] Hamdane M Dourlen P Bretteville A Sambo A-V Ferreira S Ando K. Pin1 allows for differential tau dephosphorylation in neuronal cells. Mol Cell Neurosci. (2006) 32:155–60. doi: 10.1016/j.mcn.2006.03.006, PMID: 16697218

[103] Hof PR Bouras C Buee L Delacourte A Perl DP Morrison JH. Differential distribution of neurofibrillary tangles in the cerebral cortex of dementia pugilistica and Alzheimer’s disease cases. Acta Neuropathol. (1992) 85:23–30. doi: 10.1007/bf00304630, PMID: 1285493

[104] Schmidt M Zhukareva V Newell K Lee V Trojanowski J. Tau isoform profile and phosphorylation state in dementia pugilistica recapitulate Alzheimer’s disease. Acta Neuropathol. (2001) 101:518–24. doi: 10.1007/s004010000330, PMID: 11484824

[105] McKee AC Stern RA Nowinski CJ Stein TD Alvarez VE Daneshvar DH. The spectrum of disease in chronic traumatic encephalopathy. Brain. (2013) 136:43–64. doi: 10.1093/brain/aws307, PMID: 23208308 PMC3624697

[106] Lu KP Kondo A Albayram O Herbert MK Liu H Zhou XZ. Potential of the antibody against cis–phosphorylated tau in the early diagnosis, treatment, and prevention of Alzheimer disease and brain injury. JAMA Neurol. (2016) 73:1356–62. doi: 10.1001/jamaneurol.2016.2027, PMID: 27654282

[107] Foster K Manca M McClure K Koivula P Trojanowski JQ Havas D. Preclinical characterization and IND-enabling safety studies for PNT001, an antibody that recognizes cis-pT231 tau. Alzheimers Dement. (2023) 19:4662–74. doi: 10.1002/alz.13028 37002928

[108] Marklund N. Rodent models of traumatic brain injury: methods and challenges. Methods Mol Biol. (2016) 1462:29–46. doi: 10.1007/978-1-4939-3816-2_3 27604711

[109] Vink R. Large animal models of traumatic brain injury. J Neurosci Res. (2018) 96:527–35. doi: 10.1002/jnr.24079 28500771

[110] Dai J-X Ma Y-B Le N-Y Cao J Wang Y. Large animal models of traumatic brain injury. Int J Neurosci. (2018) 128:243–54. doi: 10.1080/00207454.2017.1380008 28918695

[111] Hall GF Yao J Lee G. Human tau becomes phosphorylated and forms filamentous deposits when overexpressed in lamprey central neurons in situ. Proc Natl Acad Sci USA. (1997) 94:4733–8. doi: 10.1073/pnas.94.9.4733, PMID: 9114060 PMC20793

[112] Hall GF Lee S Yao J. Neurofibrillary degeneration can be arrested in an in vivo cellular model of human tauopathy by application of a compound which inhibits tau filament formation in vitro. J Mol Neurosci. (2002) 19:253–60. doi: 10.1385/JMN:19:3:251, PMID: 12540050

[113] Honson NS Jensen JR Abraha A Hall GF Kuret J. Small-molecule mediated neuroprotection in an in situ model of tauopathy. Neurotox Res. (2009) 15:274–83. doi: 10.1007/s12640-009-9028-y, PMID: 19384600 PMC3740339

[114] Diomede L Zanier ER Moro F Vegliante G Colombo L Russo L. Aβ1-6(D) peptide, effective on Aβ aggregation, inhibits tau misfolding and protects the brain after traumatic brain injury. Mol Psychiatry. (2023) 28:2433–44. doi: 10.1038/s41380-023-02101-3, PMID: 37198260 PMC10611578

[115] Kim W Lee S Jung C Ahmed A Lee G Hall GF. Interneuronal transfer of human tau between lamprey central neurons in situ. J Alzheimers Dis. (2010) 19:647–64. doi: 10.3233/JAD-2010-1273, PMID: 20110609

[116] Le MN Kim W Lee S McKee AC Hall GF. Multiple mechanisms of extracellular tau spreading in a non-transgenic tauopathy model. Am J Neurodegener Dis. (2012) 1:316–33. PMID: 23383401 PMC3560471

[117] Lee S Jung C Lee G Hall GF. Exonic point mutations of human tau enhance its toxicity and cause characteristic changes in neuronal morphology, tau distribution and tau phosphorylation in the lamprey cellular model of Tauopathy. J Alzheimers Dis. (2009) 16:99–111. doi: 10.3233/JAD-2009-0954 19158426

[118] McCutcheon V Park E Liu E Sobhebidari P Tavakkoli J Wen X-Y. A novel model of traumatic brain injury in adult zebrafish demonstrates response to injury and treatment comparable with mammalian models. J Neurotrauma. (2017) 34:1382–93. doi: 10.1089/neu.2016.4497, PMID: 27650063

[119] Tikhonova MA Maslov NA Bashirzade AA Nehoroshev EV Babchenko VY Chizhova ND. A novel laser-based zebrafish model for studying traumatic brain injury and its molecular targets. Pharmaceutics. (2022) 14:1751. doi: 10.3390/pharmaceutics14081751, PMID: 36015377 PMC9416346

[120] Gill T Locskai LF Burton AH Alyenbaawi H Wheeler T Burton EA. Delivering traumatic brain injury to larval zebrafish. Methods Mol Biol. (2024) 2707:3–22. doi: 10.1007/978-1-0716-3401-1_1 37668902

[121] Alyenbaawi H Kanyo R Locskai LF Kamali-Jamil R DuVal MG Bai Q. Seizures are a druggable mechanistic link between TBI and subsequent tauopathy. Elife. (2021) 10. doi: 10.7554/eLife.58744, PMID: 33527898 PMC7853719

[122] Wu BK Yuan RY Chang YP Lien HW Chen TS Chien HC. Epicatechin isolated from Tripterygium wilfordii extract reduces tau-GFP-induced neurotoxicity in zebrafish embryo through the activation of Nrf2. Biochem Biophys Res Commun. (2016) 477:283–9. doi: 10.1016/j.bbrc.2016.06.058, PMID: 27301640

[123] Lopez A Lee SE Wojta K Ramos EM Klein E Chen J. A152T tau allele causes neurodegeneration that can be ameliorated in a zebrafish model by autophagy induction. Brain. (2017) 140:1128–46. doi: 10.1093/brain/awx005, PMID: 28334843 PMC5382950

[124] Cosacak MI Bhattarai P Bocova L Dzewas T Mashkaryan V Papadimitriou C. Human TAU overexpression results in TAU hyperphosphorylation without neurofibrillary tangles in adult zebrafish brain. Sci Rep. (2017) 7:12959. doi: 10.1038/s41598-017-13311-5, PMID: 29021554 PMC5636889

[125] Bennett RE Robbins AB Hu M Cao X Betensky RA Clark T. Tau induces blood vessel abnormalities and angiogenesis-related gene expression in P301L transgenic mice and human Alzheimer’s disease. Proc Natl Acad Sci USA. (2018) 115:E1289–98. doi: 10.1073/pnas.1710329115, PMID: 29358399 PMC5819390

[126] Ramsden M Kotilinek L Forster C Paulson J McGowan E SantaCruz K. Age-dependent neurofibrillary tangle formation, neuron loss, and memory impairment in a mouse model of human tauopathy (P301L). J Neurosci. (2005) 25:10637–47. doi: 10.1523/JNEUROSCI.3279-05.2005, PMID: 16291936 PMC6725849

[127] Maheras AL Dix B Carmo OMS Young AE Gill VN Sun JL. Genetic pathways of Neuroregeneration in a novel mild traumatic brain injury model in adult zebrafish. eNeuro. (2018) 5. doi: 10.1523/ENEURO.0208-17.2017, PMID: 29302617 PMC5752677

[128] Miansari M Mehta MD Schilling JM Kurashina Y Patel HH Friend J. Inducing mild traumatic brain injury in *C. elegans* via cavitation-free surface acoustic wave-driven ultrasonic irradiation. Sci Rep. (2019) 9:12775. doi: 10.1038/s41598-019-47295-1, PMID: 31485018 PMC6726767

[129] Angstman NB Frank H-G Schmitz C. Hypothermia ameliorates blast-related lifespan reduction of *C. elegans*. Sci Rep. (2018) 8:10549. doi: 10.1038/s41598-018-28910-z 30002423 PMC6043530

[130] Angstman NB Kiessling MC Frank H-G Schmitz C. High interindividual variability in dose-dependent reduction in speed of movement after exposing *C. elegans* to shock waves. Front Behav Neurosci. (2015) 9:12. doi: 10.3389/fnbeh.2015.00012 25705183 PMC4319468

[131] Zanier ER Barzago MM Vegliante G Romeo M Restelli E Bertani I. *C. elegans* detects toxicity of traumatic brain injury generated tau. Neurobiol Dis. (2021) 153:105330. doi: 10.1016/j.nbd.2021.105330, PMID: 33711491 PMC8039186

[132] Zanier ER Bertani I Sammali E Pischiutta F Chiaravalloti MA Vegliante G. Induction of a transmissible tau pathology by traumatic brain injury. Brain. (2018) 141:2685–99. doi: 10.1093/brain/awy193, PMID: 30084913 PMC6113646

[133] Riddle DL Blumenthal T Meyer BJ Priess JR. Aging in *C. elegans*. 2nd ed. New York: Cold Spring Harbor Laboratory Press (1997). 21413221

[134] Heidary G Fortini ME. Identification and characterization of the Drosophila tau homolog. Mech Dev. (2001) 108:171–8. doi: 10.1016/S0925-4773(01)00487-7, PMID: 11578871

[135] Ubhi KK Shaibah H Newman TA Shepherd D Mudher A. A comparison of the neuronal dysfunction caused by Drosophila tau and human tau in a Drosophila model of tauopathies. Invertebr Neurosci. (2007) 7:165–71. doi: 10.1007/s10158-007-0052-4, PMID: 17636367

[136] Saikumar J Byrns CN Hemphill M Meaney DF Bonini NM. Dynamic neural and glial responses of a head-specific model for traumatic brain injury in. Proc Natl Acad Sci USA. (2020) 117:17269–77. doi: 10.1073/pnas.2003909117, PMID: 32611818 PMC7382229

[137] Sun M Chen LL. A novel method to model chronic traumatic encephalopathy in Drosophila. J Vis Exp. (2017). doi: 10.3791/55602, PMID: 28715400 PMC5608539

[138] Katzenberger RJ Loewen CA Bockstruck RT Woods MA Ganetzky B Wassarman DA. A method to inflict closed head traumatic brain injury in Drosophila. J Vis Exp. (2015) 100:e52905. doi: 10.3791/52905 PMC4544997 26168076

[139] Barekat A Gonzalez A Mauntz RE Kotzebue RW Molina B El-Mecharrafie N. Using Drosophila as an integrated model to study mild repetitive traumatic brain injury. Sci Rep. (2016) 6:25252. doi: 10.1038/srep25252, PMID: 27143646 PMC4855207

[140] Saikumar J Kim J Byrns CN Hemphill M Meaney DF Bonini NM. Inducing different severities of traumatic brain injury in Drosophila using a piezoelectric actuator. Nat Protoc. (2021) 16:263–82. doi: 10.1038/s41596-020-00415-y, PMID: 33277631 PMC8063611

[141] Huber BR Meabon JS Martin TJ Mourad PD Bennett R Kraemer BC. Blast exposure causes early and persistent aberrant phospho-and cleaved-tau expression in a murine model of mild blast-induced traumatic brain injury. J Alzheimers Dis. (2013) 37:309–23. doi: 10.3233/JAD-130182, PMID: 23948882 PMC4126588

[142] Rubenstein R McQuillan L Wang KKW Robertson C Chang B Yang Z. Temporal profiles of P-tau, T-tau, and P-tau:tau ratios in cerebrospinal fluid and blood from moderate-severe traumatic brain injury patients and relationship to 6-12 month global outcomes. J Neurotrauma. (2023) 41:0479. doi: 10.1089/neu.2022.0479 37725589

[143] Goldstein LE Fisher AM Tagge CA Zhang X-L Velisek L Sullivan JA. Chronic traumatic encephalopathy in blast-exposed military veterans and a blast neurotrauma mouse model. Sci Transl Med. (2012) 4:134ra60. doi: 10.1126/scitranslmed.3003716 PMC3739428 22593173

[144] Rice RA Pham J Lee RJ Najafi AR West BL Green KN. Microglial repopulation resolves inflammation and promotes brain recovery after injury. Glia. (2017) 65:931–44. doi: 10.1002/glia.23135, PMID: 28251674 PMC5395311

[145] Ritzel RM Li Y Jiao Y Lei Z Doran SJ He J. Brain injury accelerates the onset of a reversible age-related microglial phenotype associated with inflammatory neurodegeneration. Sci Adv. (2023) 9:eadd1101. doi: 10.1126/sciadv.add1101, PMID: 36888713 PMC9995070

[146] Simon DW McGeachy MJ Bayır H Clark RSB Loane DJ Kochanek PM. The far-reaching scope of neuroinflammation after traumatic brain injury. Nat Rev Neurol. (2017) 13:572. doi: 10.1038/nrneurol.2017.116 28776601

[147] Wang C-F Zhao C-C Liu W-L Huang X-J Deng Y-F Jiang J-Y. Depletion of microglia attenuates dendritic spine loss and neuronal apoptosis in the acute stage of moderate traumatic brain injury in mice. J Neurotrauma. (2020) 37:43–54. doi: 10.1089/neu.2019.6460, PMID: 31397209

[148] Witcher KG Bray CE Chunchai T Zhao F O’Neil SM Gordillo AJ. Traumatic brain injury causes chronic cortical inflammation and neuronal dysfunction mediated by microglia. J Neurosci. (2021) 41:1597–616. doi: 10.1523/JNEUROSCI.2469-20.2020, PMID: 33452227 PMC7896020

